# Multimorbidity of cardiovascular disease subtypes in a prospective cohort of 1.2 million UK women

**DOI:** 10.1136/openhrt-2023-002552

**Published:** 2023-12-14

**Authors:** Jae Won Suh, Sarah Floud, Gillian K Reeves, Benjamin J Cairns, F Lucy Wright

**Affiliations:** 1 Cancer Epidemiology Unit, Nuffield Department of Population Health, University of Oxford, Oxford, UK; 2 Research Department of Clinical, Educational & Health Psychology, University College London, London, UK; 3 Our Future Health, London, UK; 4 Nuffield Department of Population Health, University of Oxford, Oxford, UK; 5 Unit of Health-Care Epidemiology, Nuffield Department of Population Health, University of Oxford, Oxford, UK

**Keywords:** epidemiology, electronic health records, coronary artery disease, stroke, arrhythmias, cardiac

## Abstract

**Objective:**

Cardiovascular multimorbidity (CVM) is the co-occurrence of multiple cardiovascular disease subtypes (CVDs) in one person. Because common patterns and incidence of CVM are not well-described, particularly in women, we conducted a descriptive study of CVM in the Million Women Study, a large population-based cohort of women.

**Methods:**

UK women aged 50–64 years were followed up using hospital admissions and mortality records for an average of 19 years. CVM was defined as having ≥2 of 19 selected CVDs. The age-specific cumulative incidence of CVM between age 60 and 80 years was estimated. The numbers and proportions of individual, pairs and other combinations of CVDs that comprised incident CVM were calculated. For each individual CVD subtype, age-standardised proportions of the counts of other co-occurring CVDs were estimated.

**Results:**

The age-specific likelihood of having CVM nearly doubled every 5 years between age 60 and 80 years. Among 1.2 million women without CVD at study baseline, 16% (n=196 651) had incident CVM by the end of follow-up. Around half of all women with CVM had a diagnosis of ischaemic heart disease (n=102 536) or atrial fibrillation (n=96 022), almost a third had heart failure (n=72 186) and a fifth had stroke (n=40 442). The pair of CVDs with the highest age-adjusted incidence was ischaemic heart disease and atrial fibrillation (18.95 per 10 000 person-years). Over 60% of individuals with any given CVD subtype also had other CVDs, after age standardisation.

**Conclusions:**

CVM is common. The majority of women with any specific CVD subtype eventually develop at least one other. Clinical and public health guidelines for CVD management should acknowledge this high likelihood of CVM.

**WHAT IS ALREADY KNOWN ON THIS TOPIC**

- Specific cardiovascular disease subtypes such as ischaemic heart disease and stroke often occur together, and this is reflected in the clinical management of these conditions.
- Yet, very few studies have quantified the incidence of co-occurring cardiovascular diseases (cardiovascular multimorbidity (CVM)) beyond a few of the most common cardiovascular disease subtypes.
- Furthermore, despite known differences in the presentation of cardiovascular diseases in women compared with men, there is limited evidence on CVM specific to women.

**WHAT THIS STUDY ADDS**

- This study provides reliable estimates of the incidence of CVM in a population-based cohort of over a million UK women.
- CVM was more common than any of the 19 cardiovascular disease subtypes examined in this study.
- At least 60% of middle-aged and older women with a diagnosis of any specific cardiovascular disease subtype were eventually diagnosed with one or more other subtypes.

**HOW THIS STUDY MIGHT AFFECT RESEARCH, PRACTICE OR POLICY**

- Clinical and public health management of cardiovascular conditions in women should acknowledge that individual cardiovascular diseases are usually not isolated diagnoses.
- Understanding co-occurring cardiovascular conditions could help develop more individualised prevention and treatment.
- Future research should investigate how risk factors are shared across common patterns of CVM identified in this study.

## Introduction

Multimorbidity is commonly defined as the co-occurrence of two or more chronic conditions and is an urgent public health concern.^1^ It is estimated that between 2015 and 2035, the prevalence of multimorbidity in those aged 65 years or older will increase from 54% (n=5.3 million) to 68% (n=9.9 million) in the UK, and that the number of people with complex multimorbidity (ie, four or more chronic conditions) will double to 2.5 million.^2^

Co-occurring cardiovascular disease (CVD) subtypes (eg, coronary heart disease, stroke) and cardiovascular risk factors (eg, hypertension, hyperlipidaemia) are the most common components of multimorbidity in older adults across various populations.^3 4^ Different CVD subtypes occur together and with other chronic diseases more often than expected by chance,^5 6^ and they may share pathophysiological mechanisms that could be targeted for multimorbidity prevention.^5 7^ Furthermore, studies tracing the development of multimorbidity over time found that heart diseases, hypertension and diabetes were among the most common initial disease in people who later developed multimorbidity.^8^

Cardiovascular multimorbidity (CVM) is defined in this study as the co-occurrence of CVD subtypes. CVM is associated with a range of negative health outcomes such as higher mortality,^9^ poorer quality of life^10^ and heavier healthcare utilisation.^11^ However, most previous studies have limited the definition of CVM to only a few conditions, often focussing on ischaemic heart disease and stroke,^12–14^ and very few studies have reported the incidence of specific combinations of CVD subtypes that constitute CVM.^4 15^ Furthermore, CVD in women remains understudied,^16^ and there is currently little research on CVM in women. In a large population-based prospective cohort of UK women, we investigated the incidence of CVM using 19 CVD subtypes and examined the most common forms of CVM.

## Methods

### Study design

The Million Women Study is a population-based cohort study of 1.3 million UK women enrolled between 1996 and 2001 through the National Breast Screening Programme (online supplemental appendix A).^17^ All participants were linked by their unique National Health Service (NHS) identification number to the NHS Central Registers, which provided information on cancer registrations, hospitalisations, deaths and emigrations. Disease diagnoses and causes of death were coded using the International Classification of Diseases 10th edition (ICD-10).^18^ The end of follow-up was 31 December 2019 for these analyses.

10.1136/openhrt-2023-002552.supp1 Supplementary data

### Outcomes

Nineteen CVD subtypes were selected based on clinical importance and number of cases in the Million Women Study cohort, primarily chosen from chapter IX (diseases of the circulatory system) of the ICD-10 (online supplemental appendix B). These CVDs were broadly grouped into three disease classes: heart diseases, cerebrovascular diseases and other vascular diseases. The main outcome was CVM, defined as having two or more diagnoses from these CVD subtypes. Complex CVM (having ≥4 CVDs) was also studied as a more serious form of CVM, as in previous studies.^2 19^ Individual CVDs and CVM pairs (171 possible pairs of the selected CVDs) were studied to describe in detail the components of CVM.

An incident case of a selected CVD was defined as the first event after study recruitment. An event was identified by a record with a relevant ICD-10 code in any diagnosis field in hospital admission records, or as the underlying cause of death. The date of CVM was defined as the date of the record of the second diagnosis. This could be the same as the date of the first diagnosis if two or more were first recorded on the same day. The date of complex CVM was defined as the date of the record of the fourth diagnosis, which similarly could be the same as the date of the first, second and/or third diagnoses. The date of a CVM pair was defined as the latest of the dates of record of the two diseases in a pair (which again could be the same as the earliest of the dates of record).

### Statistical analyses

Initial analyses included 1 364 233 women recruited to the Million Women Study, after standard exclusions (online supplemental appendix C). Age-specific cumulative incidence was calculated using the Kaplan-Meier failure function for CVM and five of the most common individual CVDs at ages 60, 65, 70, 75 and 80 years. These estimates represent the probability of having a diagnosed event in anyone who had lived to each specified age. The average age at first diagnosis of each CVD subtype, CVM and complex CVM was also calculated. These analyses included prevalent CVD (ie, diagnoses of CVD subtypes before or on the date of recruitment to the study) to convey the overall picture of CVM in the whole cohort prior to investigating CVM incidence.

After excluding participants with prevalent CVD or cancer (except non-melanoma skin cancer) at study baseline, the number and proportion of incident cases of each CVD subtype, CVM and complex CVM diagnosis recorded during follow-up were calculated. The number of incident cases and proportion of individual CVD subtypes were also calculated in the subsets of women who developed CVM and complex CVM, respectively.

Age-standardised proportions of the count of other cardiovascular diagnoses that co-occurred with each individual CVD subtype were presented in stacked bar charts, and presented in categories of 0, 1, 2 and 3+. Age-standardised proportions were calculated using direct standardisation using the 2013 European Standard Population^20^ and 5-year age groups from age 45 up to 75+ years.

All unique subtypes of CVM with >1000 incident cases during follow-up were presented in an UpSet plot,^21^ which illustrates the frequency of the most common combinations of two or more co-occurring cardiovascular diseases. To further compare incidence of each possible pair of CVD subtypes (ie, CVM pair), incidence rates per 10 000 person-years were displayed in a heatmap. Participants were considered to have a CVM pair if they had a record of both conditions, regardless of whether they had records of other CVDs.

All analyses were conducted in STATA V.17 (StataCorp, College Station, Texas, USA) and R Statistical Software (V.4.2.1; R Core Team 2021).

## Results

### Cumulative incidence and average age at first diagnosis of cardiovascular multimorbidity

**Figure 1:**
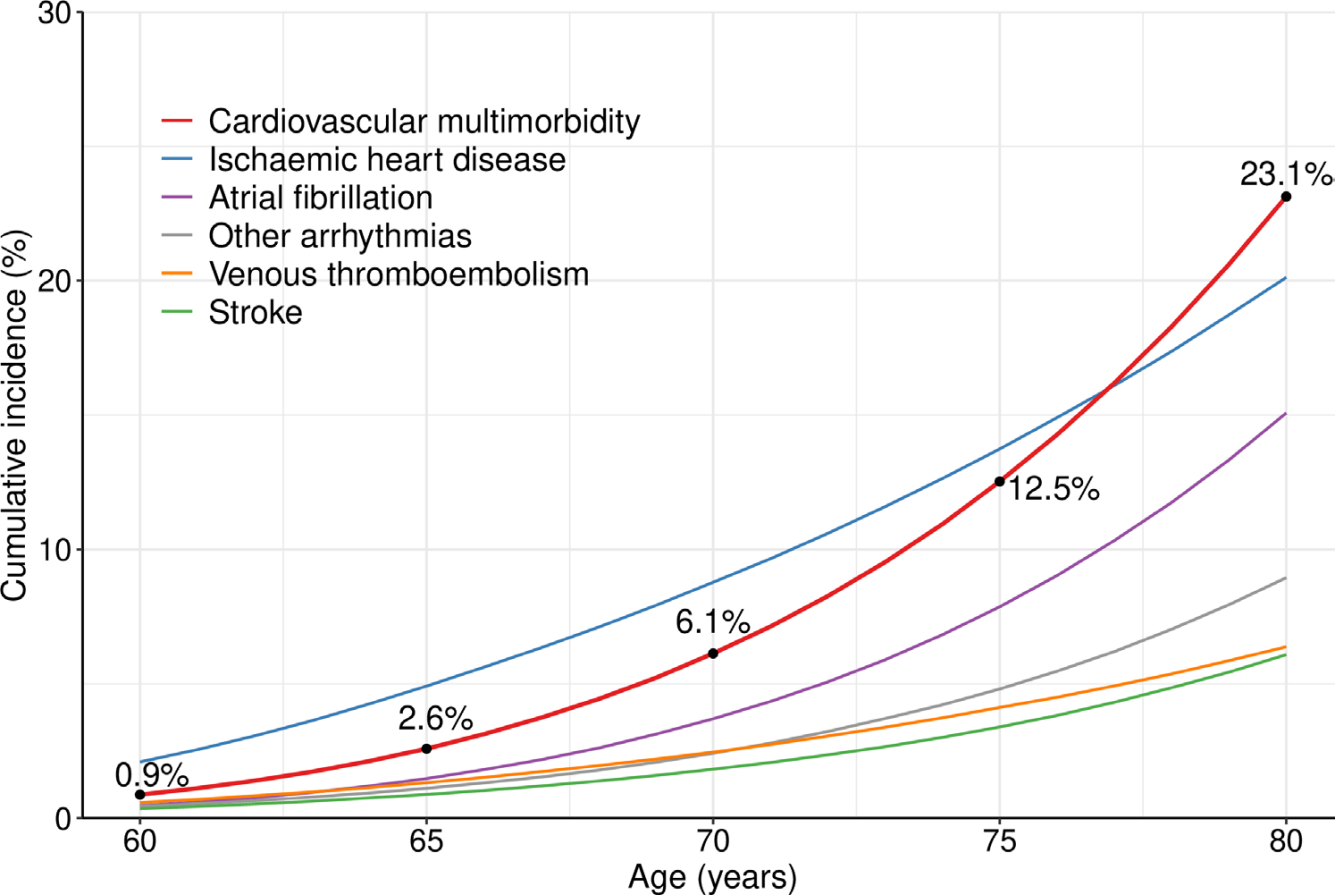
Cumulative incidence of cardiovascular multimorbidity and common individual cardiovascular diseases between age 60 and 80 years in all women.

The age-specific cumulative incidence of CVM (ie, having a diagnosis of ≥2 different CVD subtypes) nearly doubled every 5 years between ages 60 and 80 years, increasing from 0.9% to 23.1% (figure 1). The cumulative incidence of the most common individual CVD subtypes increased by 1.5-fold to 3-fold every 5 years (online supplemental appendix D). By age 80 years, women were more likely to have had CVM than ischaemic heart disease, which was the most common individual CVD subtype at any given age. Ischaemic heart disease had the earliest average age at first diagnosis (69.4 years, SD 7.8), followed by venous thromboembolism (70.1 years, SD 7.9) (table 1). In comparison, the average age at first diagnosis was 73.0 (SD 7.2) years for CVM, and 75.5 (6.6) for complex CVM (≥4 CVD subtypes).

**Table 1:** Average age of first diagnosis of cardiovascular multimorbidity and individual cardiovascular diseases in all women

Disease	ICD-10 code*	Average age of first diagnosis	SD
Cardiovascular multimorbidity			
CVM (2+ diseases)	–	73.0	7.2
Complex CVM (4+ diseases)	–	75.5	6.6
Individual cardiovascular diseases			
Vascular dementia	F01	78.3	5.6
Aortic stenosis	I35.0, I35.2	74.7	6.9
Rheumatic heart disease	I05-I09	74.6	6.9
Other cerebrovascular disease	I62, I65-69	74.4	7.3
Heart failure	I11.0, I13.0, I13.2, I50	74.1	7.3
Aortic aneurysm	I71	74.1	6.4
Other aortic valve disorders	I35.1, I35.8, I35.9	73.3	7.3
Atrial fibrillation	I48	73.3	7.0
Mitral valve disorders	I34	73.1	7.2
Other arrhythmias	I44-45, I47, I49	72.8	7.6
Vascular disorders of the intestine	K55	72.4	7.0
Stroke	I60, 61, I63, I64	72.3	7.8
Transient cerebral ischaemic attacks	G45	71.7	7.7
Peripheral vascular disease	I73.9	71.5	7.7
Cardiomyopathy	I42	71.1	7.3
Arterial embolism and thrombosis	I74	71.1	7.6
Hypertensive heart and renal disease	I12-13	70.7	6.4
Venous thromboembolism	I26, I80-82	70.1	7.9
Ischaemic heart disease	I20-25	69.4	7.8

Average age at first diagnosis for each disease was calculated using the age at first hospital admission after recruitment with a relevant ICD-10 code in any diagnosis field or death with the diagnosis as the underlying cause, whichever came first.

*Diagnostic codes used to identify diseases from hospital admissions records.

CVM, cardiovascular multimorbidity; ICD-10, International Classification of Diseases 10th edition.

### Baseline characteristics and incident cardiovascular multimorbidity

All subsequent analyses were based on incident CVDs only. A total of 1 244 482 women with no CVD or cancer at baseline were followed-up for 19.0 (SD 4.5) years, on average. Table 2 shows the baseline characteristics of these women overall and by their incident CVM status at the end of follow-up. Overall, the mean age at recruitment was 56.5 (4.8) years, 20% were current smokers, mean body mass index (BMI) was 26.1 (4.6) kg/m^2^, 19% lived in the most deprived areas (ie, the areas with the lowest quintile of the Townsend deprivation score) and 15% reported being treated for hypertension. Women who developed CVM were, on average, older at recruitment and more likely to smoke, have a higher BMI and live in the most deprived areas than those who did not develop CVM. Those who developed incident CVM were also much more likely to report being treated for hypertension, diabetes and high blood cholesterol.

**Table 2:** Baseline characteristics of women who had no record of cardiovascular disease or cancer prior to recruitment, by the number of incident cardiovascular diseases recorded by the end of follow-up

	No CVM		CVM			All women
	0 Disease	1 Disease	2 Diseases	3 Diseases	4+ Diseases	
Women, n	839,851	207,980	93,105	48,962	54,584	1,244,482
Age at recruitment, mean (SD), years	55.7 (4.5)	57.4 (4.9)	58.3 (5.0)	59.0 (5.0)	59.7 (5.0)	56.5 (4.8)
Lifestyle and anthropometric factors						
Current smoker, % (n)	18.0 (142,174)	24.3 (47,546)	26.0 (22,767)	26.7 (12,270)	28.4 (14,531)	20.4 (239,288)
Drinker of alcoholic beverages, % (n)	67.5 (563,280)	61.6 (126,879)	59.3 (54,706)	57.7 (27,963)	56.2 (30,286)	65.0 (803,114)
No strenuous physical activity, % (n)	58.3 (473,792)	63.6 (126,739)	65.5 (58,247)	66.6 (31,058)	67.8 (35,069)	60.4 (724,905)
Body mass index, mean (SD), kg/m^2^	25.8 (4.3)	26.6 (4.8)	26.9 (5.1)	27.3 (5.3)	27.6 (5.5)	26.1 (4.6)
Height, mean (SD), cm	162.0 (6.5)	162.1 (6.7)	162.2 (6.7)	162.2 (6.9)	162.3 (6.9)	162.1 (6.6)
Socio-economic factors						
Most deprived quintile, % (n)	17.3 (144,178)	21.9 (45,143)	23.3 (21,590)	24.7 (11,994)	25.9 (14,030)	19.2 (236,935)
No educational qualifications, % (n)	39.8 (326,322)	47.7 (96,373)	50.3 (45,391)	52.5 (24,854)	55.1 (29,019)	43.1 (521,959)
Reproductive factors						
Age at menarche, mean (SD), years	13.0 (1.6)	13.0 (1.6)	13.0 (1.6)	13.0 (1.7)	13.0 (1.7)	13.0 (1.6)
Age at first birth, mean (SD), years	24.0 (4.3)	23.6 (4.2)	23.6 (4.2)	23.5 (4.2)	23.5 (4.2)	23.9 (4.3)
Age at natural menopause, mean (SD), years	49.2 (4.2)	49.0 (4.4)	49.1 (4.5)	49.0 (4.6)	49.1 (4.6)	49.1 (4.3)
Premenopausal/Perimenopausal, % (n)	15.6 (130,467)	9.3 (19,169)	6.8 (6308)	5.6 (2700)	4.4 (2385)	13.0 (161,029)
Parity, mean (SD)	2.1 (1.2)	2.2 (1.3)	2.3 (1.3)	2.3 (1.4)	2.4 (1.4)	2.2 (1.2)
Ever breastfed children in parous women, % (n)	67.8 (405,802)	67.8 (98,228)	68.4 (44,056)	68.9 (22,907)	69.3 (25,484)	67.9 (596,477)
Ever use of contraceptive pills, % (n)	62.7 (522,104)	56.2 (115,465)	53.1 (48,713)	50.4 (24,266)	48.1 (25,762)	59.8 (736,310)
Ever use of hormone replacement therapy, % (n)	50.2 (417,543)	52.0 (106,718)	50.8 (46,589)	49.1 (23,613)	46.7 (25,008)	50.4 (619,471)
Morbidities						
Being treated for hypertension, % (n)	11.4 (95,737)	17.7 (36,821)	21.2 (19,684)	25.0 (12,231)	29.4 (16,063)	14.5 (180,536)
Being treated for diabetes mellitus, % (n)	1.2 (10,069)	2.7 (5613)	3.9 (3603)	5.1 (2505)	7.3 (3958)	2.1 (25,748)
Being treated for high blood cholesterol, % (n)	2.0 (16,922)	3.2 (6722)	3.9 (3644)	4.5 (2206)	5.4 (2953)	2.6 (32,447)

CVM, cardiovascular multimorbidity.

During the follow-up period, 196 651 women (15.8%) developed incident CVM (table 3). Among those who developed incident CVM (≥2 CVDs), around half had a diagnosis of ischaemic heart disease (102 536; 52%) and a similar number had a diagnosis of atrial fibrillation (96 022; 49%). About a third of women with CVM had heart failure (65 115; 33.1%). The next most common CVDs among women with CVM were other arrhythmias (59 128; 30.1%), followed by other cerebrovascular disease (49 535; 25.2%), and stroke (40 442; 20.6%). In the subset of women who developed complex CVM (≥4 CVDs), the five most common incident CVD subtypes were the same as in women with CVM, but the proportions were higher. Unlike in women with CVM, the sixth most common recorded CVD subtype found in women with complex CVM was rheumatic heart disease (20 085; 36.8%) instead of stroke (15 666; 28.7%).

**Table 3:** Number and proportion of incident cardiovascular diseases found in women who had no cardiovascular disease or cancer at recruitment, and as components of incident cardiovascular multimorbidity and complex cardiovascular multimorbidity at the end of follow-up

Disease	ICD-10 code*	In all women (n=1,244,482), % (n)	In women with CVM (n=1,96,651), % (n)	In women with complex CVM (n=54,584), % (n)
**Heart diseases**				
Rheumatic heart disease	I05-I09	2.8 (35,111)	16.7 (32,749)	36.8 (20,085)
Hypertensive heart and renal disease	I12-13	1.1 (13,875)	5.4 (10,617)	10.1 (5489)
Ischaemic heart disease	I20-25	13.3 (164,913)	52.1 (102,536)	71.0 (38,750)
Mitral valve disorders	I34	1.8 (22,319)	10.3 (20,290)	22.1 (12,053)
Aortic stenosis	135.0, I35.2	1.8 (21,890)	9.7 (19,150)	19.7 (10,765)
Other aortic valve disorders	I35.1, I35.8, I35.9	0.8 (9514)	4.3 (8405)	9.2 (4999)
Cardiomyopathy	I42	0.6 (6848)	3.2 (6269)	7.1 (3860)
Other arrhythmias	I44-45, I47, I49	6.2 (77,736)	30.1 (59,128)	46.5 (25,373)
Atrial fibrillation	I48	10.4 (129,072)	48.8 (96,022)	70.7 (38,578)
Heart failure	I11.0, I13.0, I13.2, I50	5.8 (72,186)	33.1 (65,115)	62.0 (33,861)
**Cerebrovascular diseases**				
Stroke	I60, 61, I63, I64	4.3 (53,978)	20.6 (40,442)	28.7 (15,666)
Transient cerebral ischaemic attacks	G45	1.5 (18,742)	6.8 (13,448)	10.4 (5669)
Other cerebrovascular disease	I62, I65-69	5.0 (61,859)	25.2 (49,535)	37.5 (20,450)
Vascular dementia	F01	1.1 (13,923)	5.4 (10,717)	8.6 (4696)
**Other vascular diseases**				
Venous thromboembolism	I26, I80-82	4.7 (58,056)	15.2 (29,937)	18.2 (9935)
Aortic aneurysm	I71	0.8 (10,519)	4.0 (7799)	7.4 (4057)
Peripheral vascular disease	I73.9	1.7 (20,820)	8.4 (16,501)	14.9 (8158)
Arterial embolism and thrombosis	I74	0.5 (6466)	2.8 (5576)	5.4 (2933)
Vascular disorders of the intestine	K55	0.9 (11,406)	3.6 (7017)	5.1 (2780)

CVM (ie, >1 disease); complex CVM (ie, >3 diseases).

*Diagnostic codes used to identify diseases from hospital admissions records.

CVM, cardiovascular multimorbidity; ICD-10, International Classification of Diseases 10th edition.

### Cardiovascular disease subtypes in cardiovascular multimorbidity

**Figure 2:**
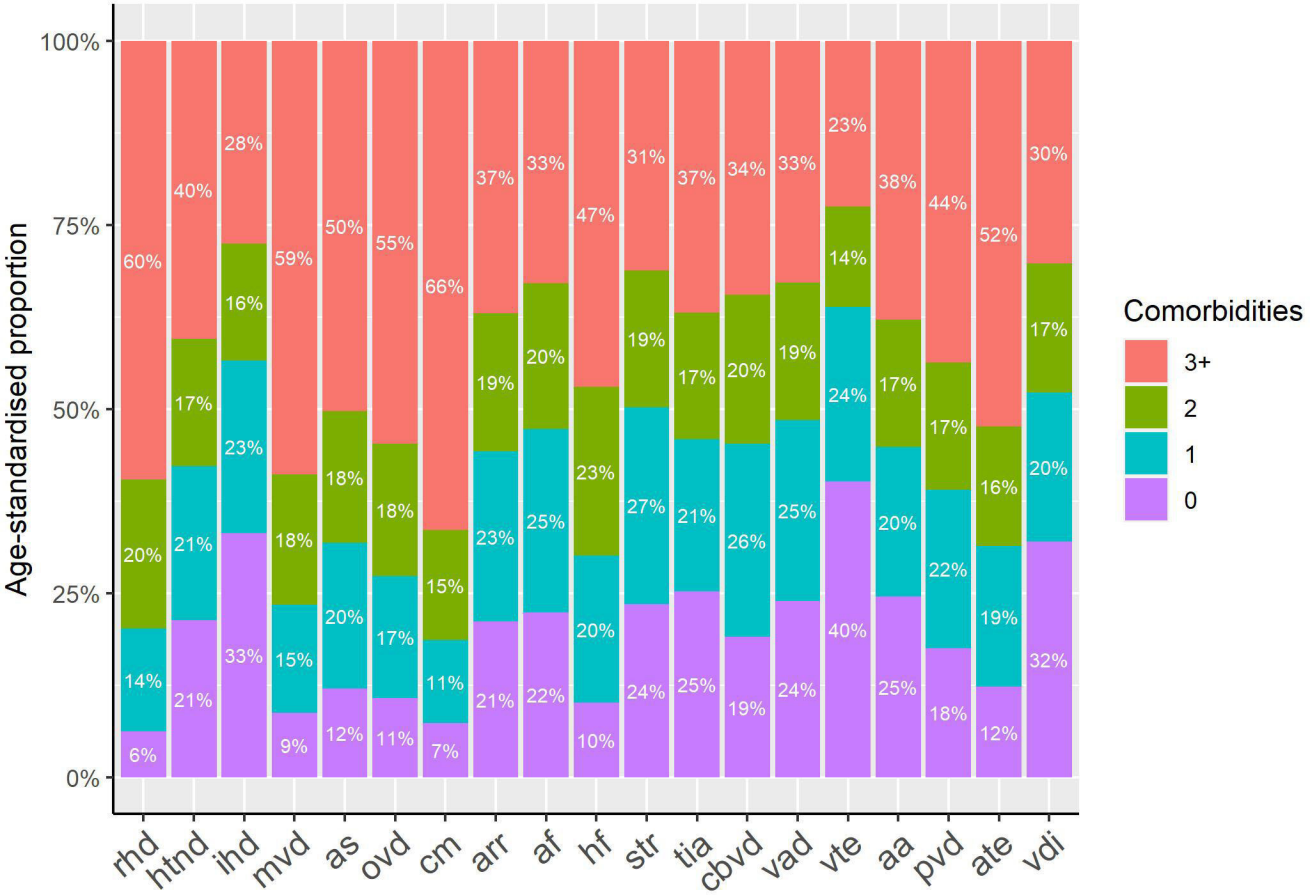
Age-standardised proportion of the number of other cardiovascular diseases by each incident individual cardiovascular disease. The x-axis displays each of the selected 19 individual cardiovascular diseases. The numbers of other cardiovascular diseases which co-occurred with each individual disease are indicated by different colours in categories of 0, 1, 2 and ≥3. For each individual cardiovascular disease, age-standardised proportions of the numbers of cardiovascular comorbidities were calculated by applying direct age standardisation to the 2013 European Standard Population, using 5-year age bands from age 45 to ≥75 years. aa, aortic aneurysm; af, atrial fibrillation; arr, other arrhythmias; as, aortic stenosis; ate, arterial embolism and thrombosis; cbvd, other cerebrovascular disease; cm, cardiomyopathy; hf, heart failure; htnd, hypertensive heart and renal disease; ihd, ischaemic heart disease; mvd, mitral valve disorders; ovd, other aortic valve disorders; pvd, peripheral vascular disease; rhd, rheumatic heart disease; tia, transient cerebral ischaemic attacks; vad, vascular dementia; vdi, vascular disorders of the intestine; vte, venous thromboembolism.

Figure 2 shows the age-standardised proportions of the number of other cardiovascular diagnoses which occurred with each individual CVD subtype during follow-up. Each bar represents participants with the CVD diagnosis specified on the horizontal axis. The colours indicate the number of other, comorbid CVD diagnoses recorded in addition to the one on the horizontal axis. Hence, ‘3+ comorbidities’ in figure 2 is equivalent to ≥4 total CVDs (ie, complex CVM). For each CVD subtype, CVM was more common than having that CVD subtype as an isolated diagnosis. The highest proportions of CVM were found in patients with rheumatic heart disease (94% with ≥1 cardiovascular comorbidities), cardiomyopathy (93%), mitral valve disease (91%) and heart failure (90%). Proportions of complex CVM (indicated as ≥3 comorbidities other than the individual CVD, ie, ≥4 total CVDs; figure 2) was especially high in participants with cardiomyopathy (66%), rheumatic heart disease (60%) and heart valve disorders (50%–59%). The highest proportions of isolated diagnoses (0 comorbidities) were found among patients with venous thromboembolism (40% of patients had the diagnosis in isolation), ischaemic heart disease (33%) and vascular disorders of the intestine (32%).

**Figure 3:**
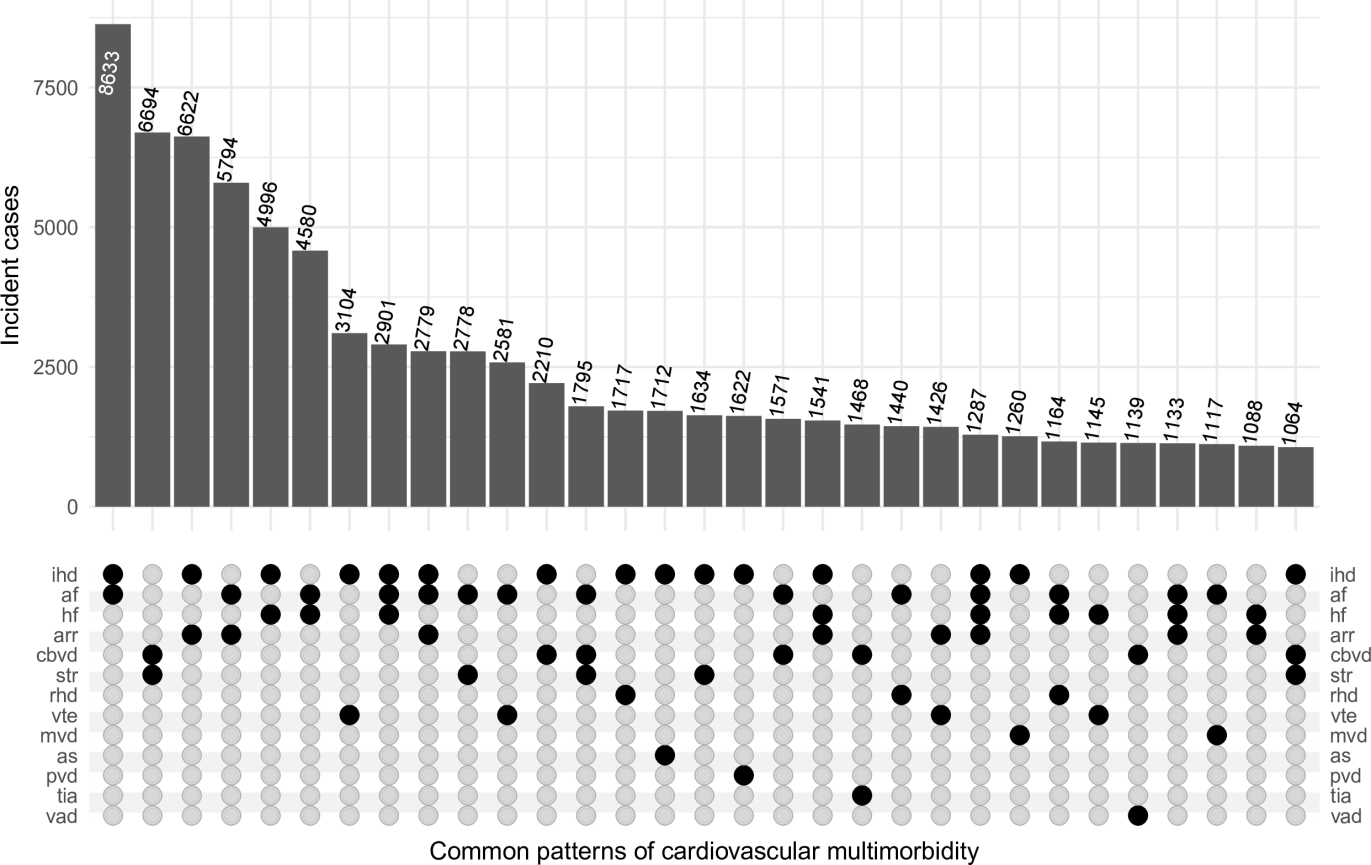
The most frequent unique combinations of two or more individual cardiovascular diseases comprising incident cardiovascular multimorbidity (n>1000). This figure displays the number of incident cases of the most common unique and mutually exclusive patterns of cardiovascular multimorbidity identified in the study cohort, in descending order from left to right. There were >1000 incident cases recorded for each pattern. Each bar displays the number of incident cases identified for the corresponding combination of cardiovascular diseases indicated in the lower panel. There are no overlaps across the bars: cases of disease combination A-B-C were not counted among the cases of disease combination A-B. af, atrial fibrillation; arr, other arrhythmias; as, aortic stenosis; ate, arterial embolism and thrombosis; cbvd, other cerebrovascular disease; hf, heart failure; htnd, hypertensive heart and renal disease; ihd, ischaemic heart disease; mvd, mitral valve disorders; pvd, peripheral vascular disease; rhd, rheumatic heart disease; tia, transient cerebral ischaemic attacks; vad, vascular dementia; vte, venous thromboembolism.

The most common unique pattern of CVM was the co-occurrence of ischaemic heart disease and atrial fibrillation (ie, without other CVD subtypes), of which there were 8633 incident cases in the cohort during the follow-up period (figure 3). Ischaemic heart disease and venous thromboembolism (n=3104), atrial fibrillation and stroke (n=2778) and atrial fibrillation and venous thromboembolism (n=2581) were the most common unique patterns of CVM with diagnoses from different disease classes (ie, heart, cerebrovascular and other vascular diseases). The most common unique combination of three CVD subtypes was ischaemic heart disease, atrial fibrillation and heart failure (n=2901), and these three diagnoses and other arrythmias formed the most common unique combination of four CVD subtypes (n=1287).

**Figure 4:**
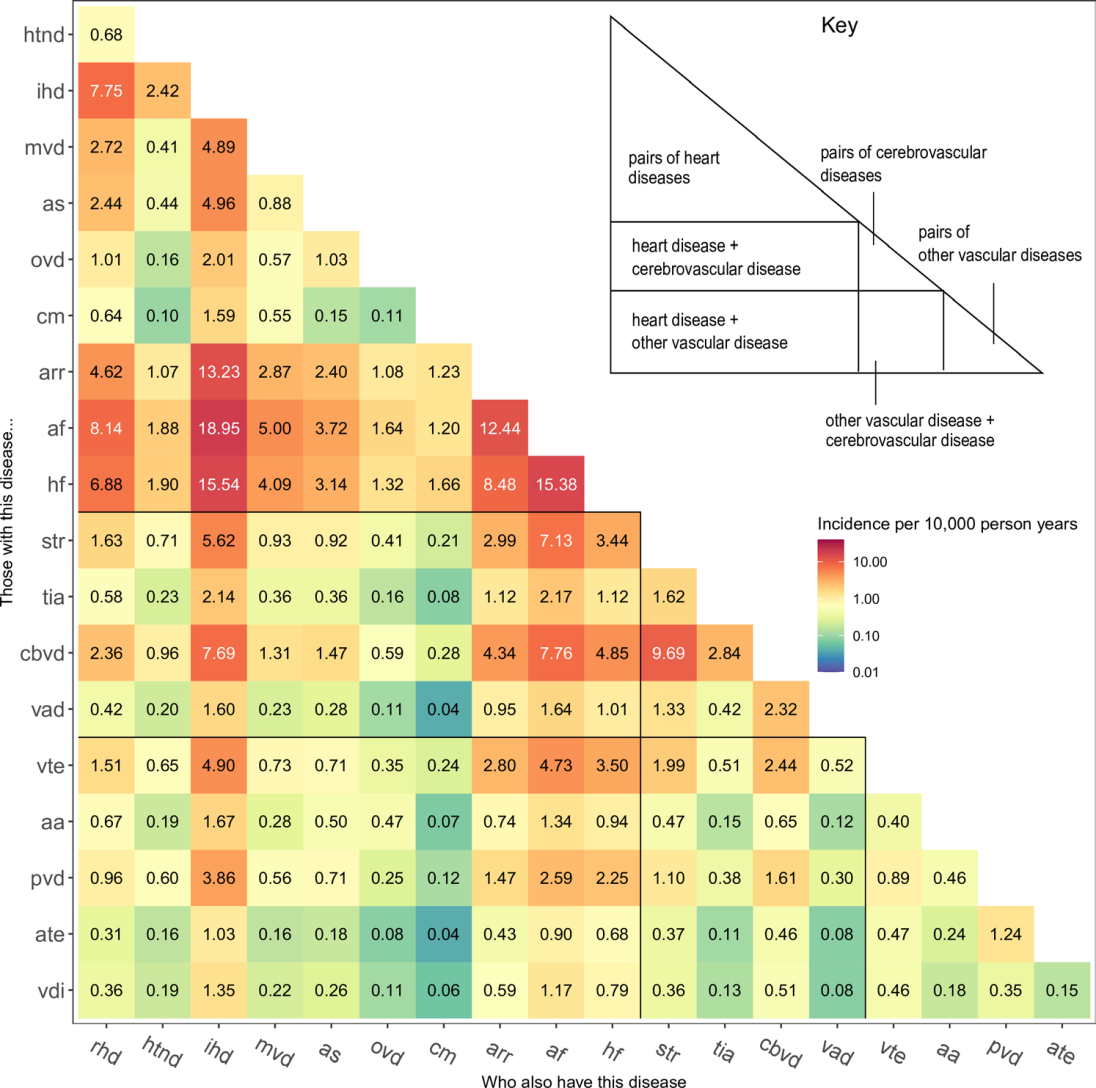
Incidence rate per 10 000 person-years for each pair of cardiovascular diseases. aa, aortic aneurysm; af, atrial fibrillation; arr, other arrhythmias; as, aortic stenosis; ate, arterial embolism and thrombosis; cbvd, other cerebrovascular disease; cm, cardiomyopathy; hf, heart failure; htnd, hypertensive heart and renal disease; ihd, ischaemic heart disease; mvd, mitral valve disorders; ovd, other aortic valve disorders; pvd, peripheral vascular disease; rhd, rheumatic heart disease; tia, transient cerebral ischaemic attacks; vad, vascular dementia; vdi, vascular disorders of the intestine; vte, venous thromboembolism.

There were 171 pairs of the 19 selected CVD subtypes. The incidence rate of the 171 CVM pairs (ie, two co-occurring CVD subtypes) ranged from 0.04 per 10 000 person-years for the pair of vascular dementia and cardiomyopathy to 18.95 per 10 000 person-years for atrial fibrillation and ischaemic heart disease (figure 4). Pairs including common heart disease diagnoses (ischaemic heart disease, atrial fibrillation, other arrhythmias and heart failure) had the highest incidence rates. The most common pair not including any of these diagnoses was stroke and other cerebrovascular disease (9.69 per 10 000 person-years). Pairs of CVDs within the same disease class were generally more common than pairs between different classes. The most common between-class pairs were the pairs of other cerebrovascular disease and atrial fibrillation (7.76), and of ischaemic heart disease and other cerebrovascular disease (7.69).

## Discussion

In 1.3 million middle-aged UK women, the age-specific risk of CVM nearly doubled between age 60 and 80 years. CVM was more common than any individual CVD subtype by age 77 years. Heart diseases (eg, ischaemic heart disease, atrial fibrillation) were the most common components of CVM, but stroke, other cerebrovascular disease and venous thromboembolism were also relatively common in CVM. The extent of CVM varied across groups of women who received a diagnosis of each individual CVD subtype, but the age-standardised proportion of CVM was over 60% in all groups. The most common unique pattern of CVM was the co-occurrence of ischaemic heart disease and atrial fibrillation, followed by the co-occurrence of stroke and other cerebrovascular disease.

### Findings in context

No studies were found that have included a comprehensive range of CVD subtypes to describe incident CVM in middle-age, despite most CVDs occurring after age 60 years in women. Few studies have previously investigated the extent and types of CVM.^4 15^ One study in US Medicare beneficiaries aged ≥65 years in 2012 investigated ischaemic heart disease, heart failure, atrial fibrillation and stroke as comorbid conditions of one another.^4^ Among 8.7 million patients with ischaemic heart disease, 36% had a diagnosis of heart failure, while 19% had a diagnosis of atrial fibrillation. Among 1.1 million patients with stroke, 58% had ischaemic heart disease and 37% had heart failure. As in our study, the US study found that the selected CVDs were common comorbidities of each other, but it was a cross-sectional analysis of prevalent rather than incident cases and did not investigate any other subtypes of CVDs.

In the UK, a study using primary care records found that among 229 205 individuals who had an incident non-fatal CVD between 2000 and 2014, the age-standardised and sex-standardised (to the 2013 European Standard Population) prevalence of cardiac arrhythmia was 7.1% (95% CI 6.0 to 9.6), heart failure was 4.1% (95% CI 3.8 to 6.3) and peripheral arterial disease was 3.8% (95% CI 3.6 to 6.0).^15^ Non-fatal CVD was defined as a diagnosis of ischaemic heart disease, stroke or transient ischaemic attack. Again, the study explored a limited selection of CVD subtypes compared with the comprehensive selection used in our study.

In our study, some CVDs had a much higher proportion with CVM than others. Rheumatic heart disease, heart valve diseases, heart failure and cardiomyopathy were found with records of comorbid CVDs around 90% or more of the time. Rheumatic heart disease, heart valve diseases and cardiomyopathy can lead to heart failure^22^; diagnoses of rheumatic heart disease also often co-occur with diagnoses of non-rheumatic heart valve disease.^23^ In general, it appears that CVD subtypes with high proportions of CVM tended to occur later in life as a result of having had other heart diseases and cardiovascular risk factors for a prolonged period of time.

One of the most common CVDs in CVM is ischaemic heart disease. However, ischaemic heart disease is among those more frequently found by itself. It shares risk factors with many other CVDs, and it is much more common than other conditions and is therefore more commonly found in isolation. The other conditions that occur more commonly in isolation include venous thromboembolism and vascular disorders of the intestine, which may have condition-specific risk factors that are less strongly implicated in the development of other CVDs. These may include major surgery, hospitalisation, active cancer and hormonal factors for venous thromboembolism,^24^ and comorbidities such as cytomegalovirus and *Escherichia coli* infection for vascular disorders of the intestine.^25^ However, there was a relatively small number of cases of vascular disorders of the intestine in this cohort, and further research is needed to better understand why some CVD subtypes are more frequently found in isolation than others.

The frequent co-occurrence and strong associations between common CVDs were anticipated, based on existing understanding of cardiovascular comorbidity. For example, the most common CVM pair and the most common unique pattern of CVM in this cohort was ischaemic heart disease and atrial fibrillation (incidence rate 18.95 per 10 000 person-years). This is consistent with existing evidence that atrial fibrillation increases the risk of myocardial infarction.^26^ Similarly, atrial fibrillation is an independent risk factor for heart failure,^27^ stroke^28^ and thromboembolism,^29^ all of which comprised common CVM pairs in this cohort.

### Strengths and limitations

The large cohort size of the Million Women Study and long follow-up time is a major strength of these analyses. There were >6000 incident cases of each of the 19 selected CVD subtypes, which allowed for a comprehensive and detailed study of the incidence of various combinations of CVDs. This study is so far unique in its focus on CVM (rather than on comorbidities of a few CVD subtypes) and the breadth and depth of the CVD subtypes investigated.

The Million Women Study was able to recruit one in four women in the target age range at the time of study recruitment, although recruitment was limited to those who attended breast screening. Women who attended breast screening were, on average, living in more affluent areas, ethnically white and more likely to be receiving hormone replacement therapy than those who did not attend screening.^30^ Nonetheless, they were comparable in age and recent prescriptions for different medications for chronic conditions (eg, heart disease, diabetes). There was, therefore, limited evidence that the differences between the participants of the Million Women Study and the general population of women would have resulted in major disparities in the types of CVM identified in this study. However, the findings may not be generalisable to the subpopulations that were under-represented in the study cohort (eg, ethnic minorities, the most socioeconomically deprived).

The only information sources for CVDs in this study were day-case and inpatient hospital admissions and death registrations, which did not include information from general practice or outpatient, emergency, non-ambulatory or long-term care. The incidence of CVM observed in this study is likely to be an underestimate of the true incidence, since these data sources will have missed some cases of CVDs that were non-fatal and did not require inpatient hospital care^31^ (see online supplemental appendix E). Another limitation is that since HES records are compiled by clinical coders for the purpose of reimbursing hospital activity, diagnostic codes may not fully reflect the nuances of a clinician’s judgement or, in some cases, the true diagnosis. For example, I12 (hypertensive renal disease) includes glomerular diseases which are ‘due to hypertension’, including some cases of chronic kidney disease; however, there may be no information available on how I12 was ascertained or differentiated from the code for chronic kidney disease (N18). Finally, we note that particularly as the follow-up time spans nearly two decades, some CVD subtypes (eg, atrial fibrillation) may have increased in incidence in recent years due to improvements in detection or other changes in clinical practice.^32^ With additional information sources on CVDs, it would be possible to have a more complete picture of the extent of CVM and its component conditions.

### Conclusion

The high proportion of CVM among individuals with any CVD subtype supports the view that CVDs are not usually isolated diagnoses, and that clinical and public health management for CVD should also consider the role of CVM (alongside other multimorbidities) in the burden of CVDs. Of particular relevance to the cohort in this study, UK women who developed CVM were, on average, older at recruitment and more likely to smoke, have a higher BMI and live in the most deprived areas than those who did not develop CVM. Each of these factors may complicate the management both of individual CVDs and of CVM. Future studies are needed to investigate associations of CVM with these and other risk factors, and how risk factors may differ between different patterns of CVM. This information could help develop individualised prevention measures for people who have a high risk of developing the common patterns of CVM identified in the present study.

## Data Availability

Data are available on reasonable request. Data may be obtained from a third party and are not publicly available. Anonymised data used in this study can be accessed by application to the investigators and to the providers of follow-up data (eg, NHS Digital). The Million Women Study Data Access Policy can be viewed at millionwomenstudy.org/data_access.
