# Correction: *Multimorbidity of cardiovascular disease subtypes in a prospective cohort of 1.2 million UK women*

**DOI:** 10.1136/openhrt-2023-002552corr1

**Published:** 2024-01-17

Suh JW, Floud S, Reeves GK, *et al*. Multimorbidity of cardiovascular disease subtypes in a prospective cohort of 1.2 million UK women. *Open Heart* 2023;10:e002552. doi: 10.1136/openhrt-2023-002552

This article has been corrected since it was first published. Author name F Lucy Wright has been corrected.

