# Supplementary material for: Multimorbidity of cardiovascular disease subtypes in a prospective cohort of 1.2 million UK women
Source: Open Heart. 2023 Dec 14;10(2):e002552. doi: 10.1136/openhrt-2023-002552 (PMC10729279; doi:10.1136/openhrt-2023-002552)
Supplement: Supplementary data [file openhrt-2023-002552supp001.pdf]

Online supplemental material to Multimorbidity of cardiovascular disease subtypes in a prospective cohort of 1.2 million UK women

Contents

Appendix A. Further details on the Million Women Study 2

Appendix B. Selection of cardiovascular disease subtypes 6

Appendix C. Study flow diagram 9

Appendix D. Cumulative incidence of cardiovascular multimorbidity and common cardiovascular disease subtypes 10

Appendix E. Reliability of individual vascular outcomes in the Million Women Study 11

Suh JW, *et al. Open Heart* 2023; 10:e002552. doi: 10.1136/openhrt-2023-002552

Appendix A. Further details on the Million Women Study

Recruitment

Recruitment for the Million Women Study was conducted via collaborating breast screening centres in 1996-2001. At the time, the NHS Breast Screening Programme invited women aged 50 to 64 years, who were registered with a general practitioner, to mammographic screening every three years. Sixty-one screening centres in England and five centres in Scotland participated in the study, covering about half of all screening centres in the UK.

The Million Women Study recruitment questionnaire was posted in the same envelope as the screening invitation sent out from each participating centre. Almost three million women were invited to join the study. Women were asked to complete the questionnaire (including the consent form) and bring it to their screening appointment if they wished to participate. Around 1.25 million women in England and 120,000 women in Scotland completed the questionnaire and joined the study, which was approximately one in four of all UK women aged 50 to 64 years in 1996-2001.

Follow-up

Participants were mainly followed-up via electronic linkage to routinely-collected NHS databases using their unique NHS number, gender, age, and postcode. The databases contained nearly complete information on emigrations, hospital admissions (day case and inpatient), and deaths. Additional linked data (e.g. primary care data) were available for subsets of participants. Only linked data for deaths, cancer registration, and hospital admissions were used in the analyses presented in this study. Information on deaths and

cancer registrations came from the Office for National Statistics (ONS), via NHS Digital in England and the Information Services Division (ISD) in Scotland (which has been part of Public Health Scotland since April 2020). Hospital admission data were acquired from the NHS Hospital Episode Statistics in England through NHS Digital, and from the Scottish Morbidity Records in Scotland through ISD Scotland. Nearly 20 years on average after recruitment, only about 1.5% of the Million Women Study cohort was lost to follow-up.

Participant characteristics

All participant characteristics reported in this study were collected through the self-reported questionnaire completed at recruitment [available from: https://www.ceu.ox.ac.uk/research/million-women-study-1/questionnaires].

Table A1. Number and proportion of missing values for key characteristics self-reported at recruitment

All women, n	1,244,482
Established cardiovascular risk factors	% (n)
**Smoking**	
Never	48.7 (605,693)
Past	26.3 (327,329)
Current <15 per day	9.8 (122,320)
Current 15+ per day	9.4 (116,968)
Missing	5.8 (72,172)
**Alcohol consumption (units/week)**	
Rarely/Never	34.7 (431,932)
1-6	40.7 (507,102)
7-14	18.7 (232,822)
15+	5.1 (63,190)
Missing	0.8 (9,436)
**Strenuous physical activity**	
Rarely/Never	46.1 (573,670)
Up to once	29.8 (370,328)
2-3 times	14.4 (179,016)
4+ times	6.1 (76,199)
Missing	3.6 (45,269)
**Body mass index (kg/m^2)**	
<20	3.9 (48,036)

20-25	41.2 (512,822)
25-30	33.6 (417,597)
30-35	11.6 (144,314)
35+	4.6 (57,248)
Missing	5.2 (64,465)
**Height (cm)**	
<155	17.4 (216,774)
155-159	14.9 (184,977)
160-164	29.5 (367,703)
165-169	22.1 (275,324)
170+	14.5 (180,324)
Missing	1.6 (19,380)
Morbidities	% (n)
**Being treated for hypertension**	
Yes	14.5 (180,536)
No	85.4 (1,063,271)
Missing	0.1 (675)
**Being treated for diabetes mellitus**	
Yes	2.1 (25,748)
No	97.9 (1,217,953)
Missing	0.1 (781)
**Being treated for high blood cholesterol**	
Yes	2.6 (32,447)
No	85.8 (1,067,746)
Missing (including 11.5% who were not asked)	11.6 (144,289)
Socio-economic factors	% (n)
**Area deprivation***	
Least deprived fifth	20.3 (253,226)
2	20.2 (251,365)
3	20.0 (248,287)
4	19.7 (245,593)
Most deprived fifth	19.0 (236,935)
Missing	0.7 (9,076)
**Educational attainment**	
1	13.2 (163,671)
2	25.9 (322,395)
3	16.4 (203,764)
4	41.9 (521,959)
5	2.6 (32,693)
Missing	13.2 (163,671)
Reproductive factors	% (n)
**Parity**	
Nulliparous	10.8 (134,969)
1-2	55.6 (692,143)
3+	33.2 (412,808)
Missing	0.4 (4,562)

**Ever use of hormone replacement therapy**	
Never	49.1 (610,743)
Ever	49.8 (619,471)
Missing	1.1 (14,268)

*Fifths of the Townsend deprivation score[1]

[1] Townsend P, Phillimore P, Beattie A. Health and Deprivation: Inequality and the North (1st ed.): Routledge; 1988.

Appendix B. Selection of cardiovascular disease subtypes

Individual cardiovascular disease subtypes (CVDs) to comprise cardiovascular multimorbidity (CVM) were selected using the International Classification of Diseases, 10th Revision (ICD-10) classification system and linked follow-up data in a four-step process to comprehensively capture serious CVDs in the Million Women Study cohort.

In the first step, each three-character code in Chapter IX (Diseases of the Circulatory System) of the ICD-10 was assessed to exclude (1) acute conditions which are not indicative of underlying chronic diseases (e.g. I46: cardiac arrest), (2) common antecedent conditions which are not typically in and of themselves serious disease states (e.g. I10: primary hypertension), and (3) non-specific codes (e.g. I27: other pulmonary heart disease). Non-specific codes that may represent important classes of conditions that could be grouped with a specific condition (e.g. I09: other rheumatic heart diseases) were not excluded.

In the second step, CVDs which are not included in Chapter IX of ICD-10 were identified and selected. Transient cerebral ischaemic attacks and related syndromes (G45), vascular dementia (F01), and vascular disorders of intestine (K55) were included from other chapters of the ICD-10.

In the third step, the three-character ICD-10 codes selected in the first two steps were further aggregated (e.g. I20-I25: ischaemic heart disease) or separated (e.g. I35.0/2 aortic stenosis) to form aetiologically and/or anatomically distinct diseases.

In the fourth and final step, individual CVDs which were relatively common in the Million Women Study cohort were included for investigation as components of CVM in this study.

Suh JW, *et al. Open Heart* 2023; 10:e002552. doi: 10.1136/openhrt-2023-002552

An event was defined as the first hospital admission after recruitment with a relevant ICD-10 code in any position (i.e. primary or secondary diagnosis fields) or death with the diagnosis as the underlying cause, whichever came first. Only events that were recorded after the date of recruitment up to 31 December 2016 were counted. Note that these were not strictly incident events, since participants with prior CVD at recruitment had not been excluded yet. Note also that end of follow-up when the CVDs were selected was 31 December 2016, whereas the analyses presented in this study included additional follow-up time up to 31 December 2019. Nineteen CVDs with 5,000 or more events by the end of 2016 were finally included (Table B1). The selected CVDs could be broadly grouped into three disease classes: heart diseases, cerebrovascular diseases, and other vascular diseases.

We acknowledge that some of the CVD subtypes we have defined may not be clearly distinct aetiologically and/or anatomically. For example, the ICD-10 codes that define ‘other cerebrovascular disease’ encompasses a range of conditions such as ‘other nontraumatic intracranial haemorrhage (I62)’, ‘occlusion and stenosis of precerebral/cerebral arteries, not resulting in cerebral infarction (I65, I66)’ or ‘cerebral disorders in diseases classified elsewhere (I68)’ (e.g. I68.0, cerebral amyloid angiopathy). We acknowledge that these cannot be cleanly separated from stroke and vascular dementia and will largely co-occur with those conditions (which we observe in the data). However, we aimed to include a comprehensive range of CVDs as represented by the ICD-10 coding system and commonly recorded in HES and death records. We therefore chose to include these codes under the umbrella term, ‘other cerebrovascular disease’.

Table B1 List of selected individual cardiovascular diseases, corresponding diagnostic codes, and number of events between study recruitment and 31 December 2016, after standard exclusions only

	ICD-10 code*	Events (N)**
**Heart diseases**		
Rheumatic heart disease	I05-I09	22,731
Hypertensive heart and renal disease	I12-13	14,329
Ischaemic heart disease	I20-25	154,991
Mitral valve disorders	I34	16,292
Aortic stenosis	135.0, I35.2	14,801
Other aortic valve disorders	I35.1, I35.8, I35.9	6,760
Cardiomyopathy	I42	5,162
Other arrhythmias	I44-45, I47, I49	54,398
Atrial fibrillation	I48	91,980
Heart failure	I11.0, I13.0, I13.2, I50	50,089
**Cerebrovascular diseases**		
Stroke	I60, 61, I63, I64	40,530
Transient cerebral ischaemic attacks and related syndromes	G45	14,918
Other cerebrovascular disease	I62, I65-69	40,995
Vascular dementia	F01	7,810
**Arterial, venous, and other vascular diseases**		
Venous thromboembolism	I26, I80-82	35,039
Aortic aneurysm	I71	7,487
Peripheral vascular disease	I73.9	17,008
Arterial embolism and thrombosis	I74	5,069
Vascular disorders of the intestine	K55	7,994

*Diagnostic codes used to identify diseases from hospital admission and death records

**An event was defined as the first hospital admission after recruitment with a relevant ICD-10 code in any position or death with the diagnosis as the underlying cause, whichever came first. These were not considered incident events, since women with cardiovascular disease prior to recruitment were not excluded from the cohort yet.

Appendix C. Study flow diagram

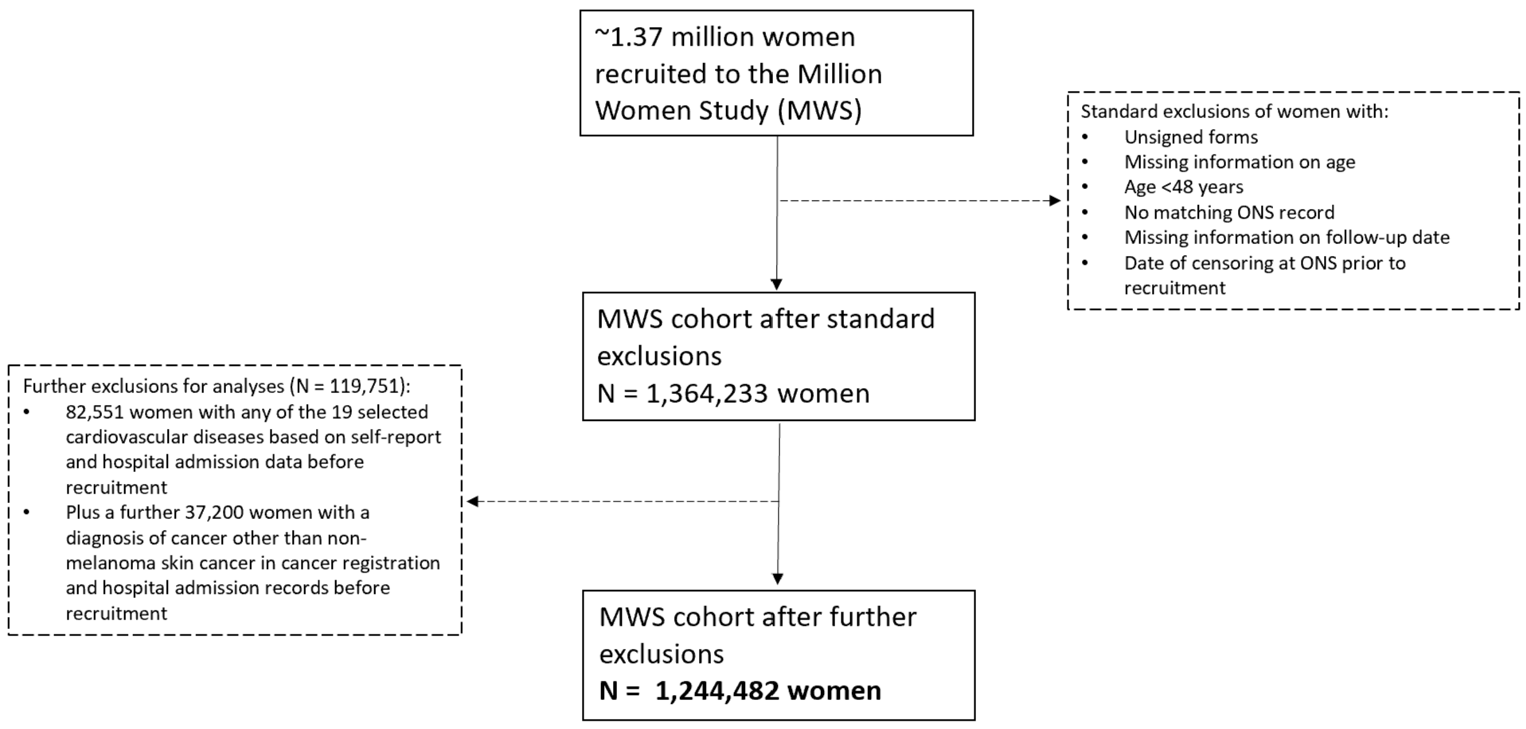

Appendix D. Cumulative incidence of cardiovascular multimorbidity and common cardiovascular disease subtypes

Cumulative incidence (%)	Age, years				
	60	65	70	75	80
Cardiovascular multimorbidity	0.9	2.6	6.1	12.5	23.1
Ischaemic heart disease	2.1	4.9	8.8	13.7	20.1
Venous thromboembolism	0.6	1.3	2.5	4.1	6.4
Other arrhythmias	0.4	1.1	2.4	4.8	8.9
Atrial fibrillation	0.5	1.5	3.7	7.9	15.1
Stroke	0.4	0.9	1.8	3.4	6.1

Suh JW, *et al. Open Heart* 2023; 10:e002552. doi: 10.1136/openhrt-2023-002552

Appendix E. Reliability of individual vascular outcomes in the Million Women Study

The reliability of vascular outcomes identified through HES compared with general practice records has been assessed by a previous study in the Million Women Study for coronary heart disease (ICD-10 codes I20-I25), cerebrovascular disease (G54, I60-I69), and venous thromboembolism (I26, I80-I82)[2]. Among a random sample of 2,323 women with a HES diagnosis of any of the three vascular outcomes, 93% (n=2,157) also had a record of diagnosis in general practice records. Among 864 women with no HES diagnosis of vascular disease, 97% (n=838) also had no record in general practice records. Agreement between HES and general practice records was highest for serious and specific outcomes such as myocardial infarction and pulmonary embolism. The 3% (n=26) who did not have a hospital admission record of vascular disease but did have a diagnosis in general practice records had less serious forms of disease; none had a diagnosis of myocardial infarction, confirmed stroke, or pulmonary embolism.

Based on these findings, the authors estimated that a substantial number of less severe vascular diseases were missed by hospital admission records. Approximately 35,000 cases of vascular disease in the study cohort were estimated to have been missed in HES data (relative to general practice records), in addition to the 61,000 cases which were identified in HES between 1997-2005. The number of chronic and less severe cardiovascular conditions is likely to be proportionately underestimated in this study.

[2] Wright FL, Green J, Canoy D, Cairns BJ, Balkwill A, Beral V, et al. Vascular disease in women: comparison of diagnoses in hospital episode statistics and general practice records in England. BMC Med Res Methodol. 2012;12:161.
